# Are Social Inequalities Widening in Generalised and Abdominal Obesity and Overweight among English Adults?

**DOI:** 10.1371/journal.pone.0079027

**Published:** 2013-11-08

**Authors:** Denise Howel, Elaine Stamp, Thomas J. Chadwick, Ashley J. Adamson, Martin White

**Affiliations:** 1 Institute of Health & Society, Newcastle University, Newcastle upon Tyne, United Kingdom; 2 Human Nutrition Research Centre, Newcastle University, Newcastle upon Tyne, United Kingdom; 3 Fuse, UKCRC Centre for Translational Research in Public Health, Newcastle upon Tyne, United Kingdom; Indiana University, United States of America

## Abstract

**Background:**

Obesity is now more common in lower socioeconomic groups in developed nations, but the socio-economic patterning of obesity has changed over time. This study examines the time trends in the socioeconomic patterning of generalised and abdominal obesity and overweight in English adults.

**Methods:**

Data were from core annual samples of the Health Survey for England 1993–2008, including 155 661 participants aged 18–75 years. The prevalence of generalised and abdominal obesity and overweight was reported as crude and age-adjusted estimates. Binomial regression was used to model measures of obesity and overweight with age, sex, survey years, and two indicators of socioeconomic position: Registrar General’s Social Class (manual and non-manual occupational groups) and relative length of full time education. Trends in socioeconomic patterning were assessed by formal tests for interactions between socioeconomic position measures and survey periods in these models.

**Results:**

The prevalence of generalised and abdominal overweight and obesity increased consistently between 1993 and 2008. There were significant differences in the four outcomes between the two socioeconomic position (SEP) groups in men and women, except for generalised and abdominal overweight with social class in men. The prevalence of obesity and overweight across the whole period was higher in subgroups with lower SEP (differences of 0.2% to 9.5%). There was no significant widening of the socioeconomic gradient of most indicators of greater body fat since the early 1990s, except for educational gradient in generalised obesity in men and women (P = 0.001).

**Conclusions:**

Substantial social class and education gradients in obesity and overweight are still present in both sexes. However, there is limited evidence that these socioeconomic inequalities have changed since 1993.

## Background

The considerable increase in the prevalence of obesity and overweight over the last three decades has now reached epidemic proportions [1], though there are some signs that the epidemic is levelling off [2]. As obesity has become more common, the socioeconomic groups most affected by obesity have changed. In the first half of the 20th century, obesity was a disease of affluence, but in more recent decades, it has been seen more often in lower socioeconomic groups. This pattern emerged first in high-income countries, and more recently in low- and middle-income countries [3].

There has been interest in both cross-sectional patterns of obesity by socioeconomic position (SEP) and any patterns in time-trends by SEP. The cross-sectional relationship between SEP and obesity was summarised in a recent international review which found that, in developed countries, women in lower SEP groups had a greater likelihood of obesity; though, for men, the relationship was less consistent. [4] When repeated cross-sectional data have been available, a number of studies have investigated whether the increasing time-trend in obesity and other measures of body size has occurred equally in all SEP categories. However, these have not provided a clear consensus, finding that socioeconomic inequality has increased, decreased or remained unchanged [5]–[24].

National Health Surveys have been undertaken annually in England since 1991 and provide the most comprehensive available data at a national level on a range of health and social variables, including measures of body shape and weight. Although limited by its cross-sectional design, the Health Survey for England (HSE) provides a lens through which changing health and risk factors in the English population can be viewed and evaluated [25]. Using the HSE data between 1993 and 2008, this study aimed to investigate the trends in socioeconomic patterning of obesity and overweight in adults in England, based on cut-offs for both body mass index (BMI) and waist circumference (WC). Interest was concentrated on the average difference over the whole period in the prevalence of obesity and overweight between subgroups defined by occupation and education, and whether any social inequalities in obesity and overweight had changed over time.

## Methods

### Setting and Study Design

We investigated social trends in obesity and overweight over time by bringing together all core annual cross-sectional samples from the HSE from 1993 to 2008, providing BMI and WC data on up to 155 661 participants aged 18–75 years. Households were identified using multi-stage sampling from a comprehensive listing of postal addresses in a geographically representative manner: all adults in a household were invited for interview. A new sample of people was invited every year. Socio-demographic information and height and weight measurements were collected by standard procedures by trained interviewers at the homes of participants. Weight was measured using Soehnle, Seca and Tanita electronic scales. Participants were asked to remove any shoes or bulky clothing, and were not weighed if they were pregnant, unsteady on their feet or chair-bound. A single measurement was recorded to the nearest 100 g. Height was measured using a portable stadiometer with a sliding head plate, a base plate and three connecting rods marked with a metric measuring scale. Participants were asked to remove their shoes. One measurement was taken to the nearest millimetre. WC was defined as the midpoint between the lower rib and upper margin of the iliac crest, measured by a nurse using a tape with an insertion buckle at one end. The measurement was taken twice and recorded to the nearest even millimetre. The response rates varied from year to year, but, overall, around 70% of survey participants agreed to an interview, BMI was available on around 90% of those interviewed, and waist circumference on around 80%. Details of sampling procedures and data collection methods have been published previously. [26] The analysis only used data in the core samples collected each year, and not the boost samples which concentrated on particular subgroups in some years. The analysis was restricted to the age range 18–75, within which obesity and overweight generally rise with age. In addition, any relationships in older ages are complicated, in that those who survive longer are not typical of the general population.

### Measures

The outcome measures used in analyses were: generalised obesity and overweight based on BMI categories (BMI≥30.0 kg/m^2^ and BMI≥25.0 kg/m^2^ respectively), as defined by WHO; [1] and WC categories for abdominal obesity and overweight (WC≥102 cm and ≥94 cm respectively in men, and ≥88 cm and ≥80 cm respectively in women). These are the category 1 and 2 cut-offs for WC used as the definition of abdominal obesity in white European origin adults for metabolic syndrome by the Adult Treatment Panel III [27] and the International Diabetes Federation [28]. Note that overweight categories also include those who are obese. WC was not collected in 1995–1996, 1999–2000 and 2004. Covariates were age, sex, year of survey and two measures of socio-economic position (SEP) based on the Registrar General’s Social Class [29], [30] and relative length of full-time education.

All members of participating households were assigned the social class of the head of their household using the Standard Occupational Classification (SOC) 1990 for the years 1993–1999 and the SOC 2000 for subsequent years. Registrar General’s Social Class was re-coded into non-manual and manual occupational groups for analyses: this information was missing for 941 participants. Participants where the head of household’s occupation fell into other categories (armed forces, full-time students, never worked and unclassified, (n = 4591)) were excluded from the analysis due to the small numbers in each category. The proportion of participants where the head of household’s occupation was coded as manual declined from 49% in 1993 to 40% in 2008: there was a noticeable drop in 2000, most likely due to coding changes from SOC 1990 to SOC 2000.

Educational history was described in relative, rather than absolute, terms. It identified those with a higher or lower education-based SEP relative to their age, rather than simply the amount of education they had received. The length of full-time education was split into two categories by age group; ‘longer’ or ‘shorter’, while taking into account that longer education is more common in those born more recently. The ‘shorter’ category was defined as those leaving at or earlier than the median age of leaving full time education for each age group (the medians being 18 yrs for those aged 19–24, 16 yrs for ages 25–39 and 40–59, 15 yrs for ages 60–75). This variable was missing on 2407 participants, including those aged 18, since it was only defined on participants aged 19 or over. The proportion of participants in the ‘shorter’ category declined gradually from 66% in 1993 to 48% in 2008.

### Statistical Analysis

The analysis was split into a descriptive element and formal comparisons. The descriptive element reports estimated or graphed the prevalence of the four outcomes (generalised obesity and overweight, and abdominal obesity and overweight) overall or by SEP subgroup. Some crude estimates of prevalence have been reported to illustrate the burden of obesity and overweight in the sample, but these estimates were also age-adjusted by direct standardisation, based on the age distributions of men and women in the HSE sample in 1993/4. The formal comparisons were of the average difference over the whole period in the prevalence of obesity and overweight between subgroups defined by occupation and education, and whether any social inequalities in obesity and overweight had changed over time. In these comparisons, the relationship between the prevalence of the four binary outcomes (generalised obesity and overweight, abdominal obesity and overweight) with the explanatory variables (age, survey years and social class or educational group) was fitted by generalized linear models with binomial errors and an identity link function. This approach estimated the absolute differences in prevalence between subgroups. The survey years were grouped into four periods (1993–6, 1997–2000, 2001–4, 2005–8) and age was fitted as polynomial terms (linear, quadratic and square root). Separate models were fitted for the four outcome measures in both men and women, and with and without the linear interaction terms between survey periods and either social class or educational group. Wald tests were carried out to check for statistical significance of groups of variables encompassing all levels of a factor such as survey periods, or the interaction between survey periods and social class. Main effects were considered statistically significant if P<0.05, and interactions if P<0.01. Models that only included the main effects of factors provided an estimate of the average difference in prevalence between SEP subgroups over the whole time period. For models including the interaction between SEP factors and survey periods, the particular interaction terms have been reported that describe the change from 1993/6 to 2005/8 between prevalence differences in SEP groups. All statistical analysis was undertaken in the statistical package STATA version 12 [31].

## Results

For each combination of gender and outcome measure, the results reported in the text below are the crude prevalence in 1993/4 and 2007/8. All outcomes increased in prevalence over the period studied. Overall, generalised obesity in those aged 18–75 years rose from 13.9% in 1993/4 to 25.1% in men and from 17.4% to 25.4% in women. The prevalence of generalised overweight rose from 59.2% to 67.5% in men and from 49.2% to 57.3% in women over the same period. Abdominal obesity rose from 21.4% to 35.1% in men, and from 27.2% to 43.7% in women, while abdominal overweight rose from 47.3% to 60.1% in men, and from 50.5% to 66.5% in women. Note that ‘overweight’, as defined in the *Methods* section, is those above the ‘overweight’ thresholds, thus combining those in ‘overweight’ and ‘obese’ categories. To allow consideration of the effect of adjustment for age, the final column of Table 1 shows the prevalence of each outcome in 2007/8 age-adjusted to the 1993/4 age distribution: the difference between crude and age-adjusted prevalence in 2007/8 was very small.

**Table 1:** Trends in age-adjusted prevalence of generalised and abdominal obesity and overweight and socio-demographic factors during 1993–2008 in England.

*Years*:	1993/4	1995/6	1997/8	1999/00	2001/02	2003/04	2005/6	2007/8
MEN								
Prevalence generalised obesity (%)^1^	13.9	16.2	17.6	20.3	21.8	23.2	24.3	24.9
Prevalence generalised overweight (%)^2^	59.2	61.1	63.5	64.7	67.8	67.7	68.5	67.3
Sample size	13 145	12 688	9437	5871	8402	7636	7598	7654
Prevalence abdominal obesity (%)^3^	21.3	–	24.0	–	30.1	31.7	32.7	33.9
Prevalence abdominal overweight (%)^4^	47.3	–	49.8	–	57.1	58.9	59.0	59.1
Sample size	11 922	10	8663	10	7315	4651	6201	6028
Socio-demographic factors								
Average age (yrs)^7^	43.8	44.8	46.1	44.7	45.2	46.4	46.9	46.8
% Manual Occupation^8^	51.7	51.1	51.4	48.3	46.8	45.0	42.8	44.1
% Shorter education^9^	65.3	63.3	62.2	59.3	57.3	55.2	51.6	48.6
WOMEN								
Prevalence generalised obesity (%)^1^	17.4	18.5	21.1	21.3	23.8	23.6	24.7	25.2
Prevalence generalised overweight^2^	49.2	51.7	53.3	54.4	56.6	56.5	56.5	56.9
Sample size	14 541	14 370	10 738	6645	9820	9145	8888	9083
Prevalence abdominal obesity (%)^5^	27.2	–	30.3	–	37.6	40.4	41.0	42.6
Prevalence abdominal overweight (%)^6^	50.6	–	53.0	–	61.2	64.1	63.7	65.3
Sample size	13 174	10	9848	10	8589	5575	7465	7309
Socioeconomic factors								
Average age (yrs)^7^	44.1	44.5	44.6	45.0	45.1	46.1	46.1	46.3
% Manual Occupatio^8^	49.1	48.0	48.3	45.0	43.7	41.7	40.4	36.6
% Shorter education^9^	65.1	64.1	61.7	59.1	56.3	53.5	50.8	48.2

Definitions of obesity and overweight cut-offs:

1 BMI≥30.0 kg/m^2^.

2 BMI≥25.0 kg/m^2^.

3 WC≥102 cm.

4 WC≥94 cm.

5 WC≥88 cm.

6 WC≥80 cm.

7 Average age of HSE sample in 18–75 age-band.

8 % where head of household’s occupation coded as manual.

9 % with shorter education relative to their age-group.

10 Waist circumferences were not collected in 1995–1996, 1999–2000 and 2004.

In addition, Table 1 shows the age-adjusted prevalence of the four obesity and overweight outcomes for all 2-year periods, along with key socio-demographic variables. Over the period of this study it can be seen that the average age in the sample has increased by 3.0 years in men and 2.2 years in women, the proportion coded as ‘manual occupation’ has decreased by 7.6% in men and by 12.5% in women, and the proportion coded as ‘shorter education’ has decreased by 16.7% in men and 16.9% in women.

### Trends in Obesity, Overweight Over Time by Social Class and Relative Length of Full-time Education

**Figure 1:**
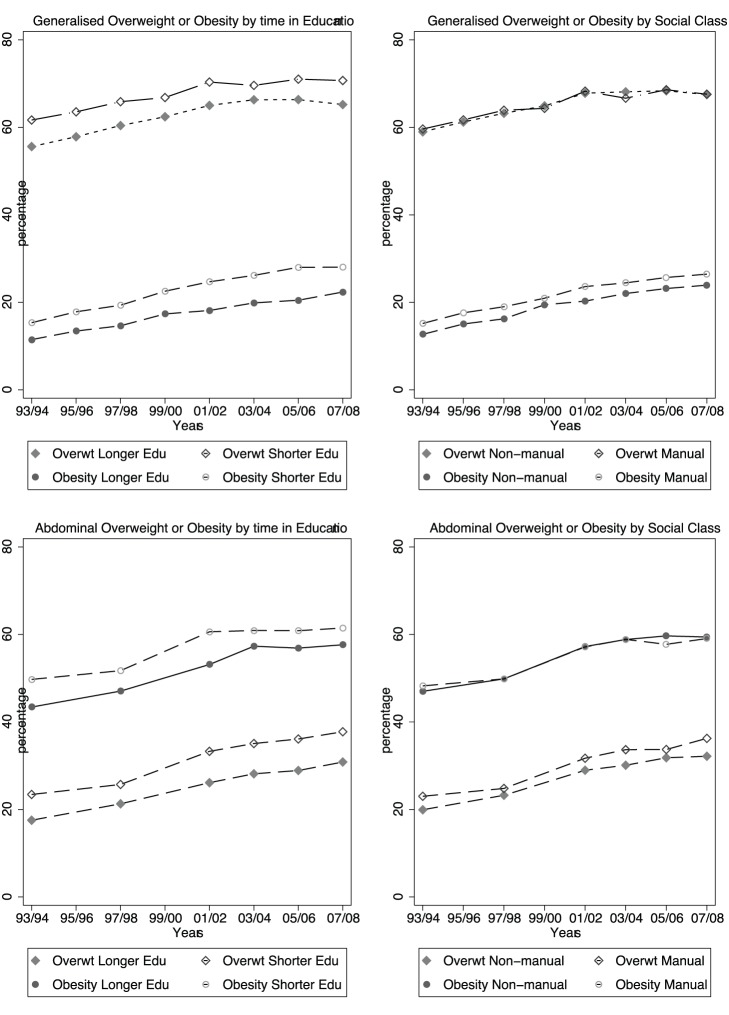
Prevalence of age-adjusted generalised obesity, generalised overweight, abdominal obesity and abdominal overweight by social class and education subgroups in men.

**Figure 2:**
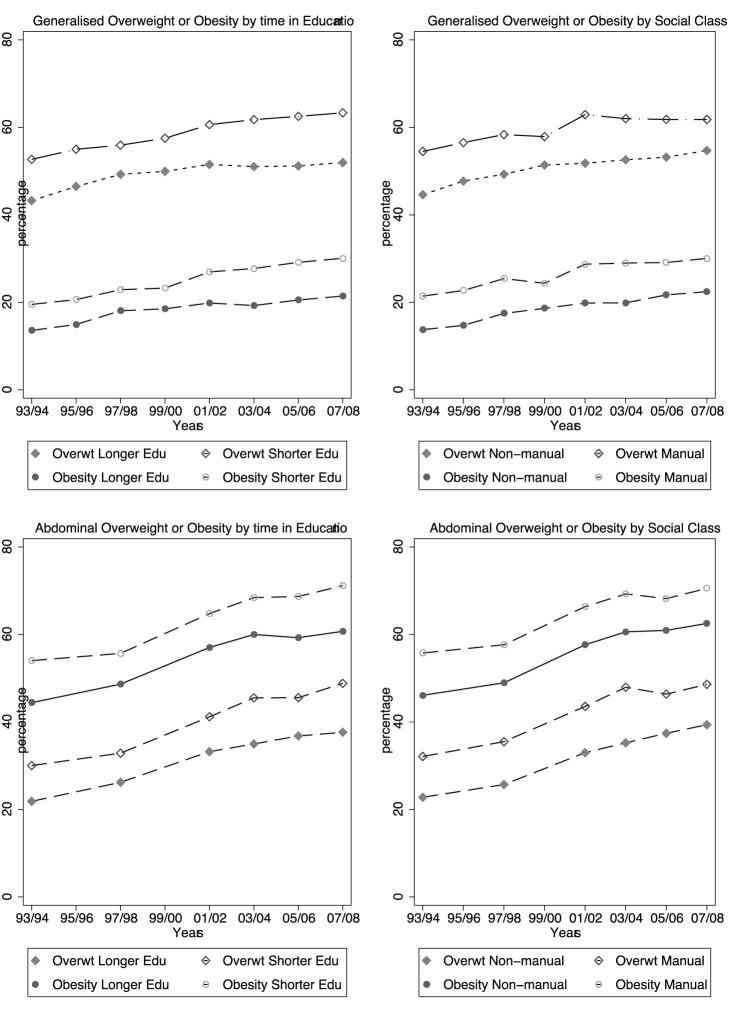
Prevalence of age-adjusted generalised obesity, generalised overweight, abdominal obesity and abdominal overweight by social class and education subgroups in women.

Figure 1 shows the trends over time, firstly in the age-adjusted prevalence of generalised obesity and overweight by social class and educational groups, and then age-adjusted prevalence of abdominal obesity and overweight by educational and social class groups for men. Figure 2 shows the same results for women. It can be seen that the lower compared to higher SEP group (as determined by education or social class) had greater prevalence of all outcomes for men and women throughout the study period, and there was no obvious widening or narrowing of the social gradients. The direction of the difference in prevalence between lower and higher SEP groups was consistent across gender, both measures of SEP, and all obesity and overweight outcomes: however the size of the difference varied.

Table 2 shows key results of the binomial regression analyses, concentrating on the main effects of SEP indicators and their interaction with survey period. Among men, all outcomes were higher in the lower SEP groups when averaged across the whole study period (difference in prevalence varying between 0.2% and 4.6%). It can be seen that these differences were statistically significant for all outcomes when comparing educational subgroups, but only for the two obesity outcomes in men when comparing social class subgroups. There was a tendency for the difference to be smaller when comparing SEP groups defined by social class rather than education, though no formal comparisons were made.

**Table 2:** Results of binomial regressions of prevalence of obesity and overweight by SEP indicator and survey period.

Prevalence measure	SEP Indicator	Sample size	Overall difference in prevalence (%)between SEP groups(95% CI)^1^	P-value forOveralldifference inprevalence	Change in prevalence (%) difference between SEPgroups from 1993/6 to2005/8^2^	P-value for interaction between SEP and survey periods^2^
MEN						
Generalised obesity^3^	Education^6^	71273	4.4 (3.9 to 5.0)	<0.0001	3.0 (1.4 to 4.6)	<0.0001
	Social class^7^	70402	2.3 (1.7 to 2.8)	<0.0001	0.9 (−0.9 to 2.5)	0.28
Generalised overweight^3^	Education	71273	4.5 (3.8 to 5.2)	<0.0001	−1.6 (−3.5 to 0.2)	0.07
	Social class	70402	0.3 (−0.4 to 0.9)	0.45	0.1 (−1.7 to 1.9)	0.93
Abdominal obesity^4,5^	Education	44794	4.6 (3.8 to 5.3)	<0.0001	2.6 (0.5 to 4.6)	0.01
	Social class	44422	2.3 (1.6 to 3.0)	<0.0001	1.0 (−1.0 to 3.1)	0.33
Abdominal overweight^4,5^	Education	44794	4.2 (3.3 to 5.1)	<0.0001	−2.7 (−5.1 to −0.4)	0.02
	Social class	44422	0.2 (−0.6 to 1.1)	0.59	−1.8 (−4.1 to 0.5)	0.13
WOMEN						
Generalisedobesity	Education	81981	6.0 (5.4 to 6.5)	<0.0001	2.7 (1.2 to 4.3)	<0.0001
	Social class	79727	7.5 (6.9 to 8.1)	<0.0001	0.2 (−1.4 to 1.8)	0.85
Generalised overweight	Education	81981	8.2 (7.5 to 8.9)	<0.0001	0.7 (−1.1 to 2.5)	0.44
	Social class	79727	8.9 (8.2 to 9.5)	<0.0001	−1.6 (−3.4 to 0.2)	0.09
Abdominalobesity	Education	52058	7.3 (6.5 to 8.1)	<0.0001	1.9 (−0.2 to 4.0)	0.08
	Social class	50976	9.5 (8.8 to 10.3)	<0.0001	0.4 (−1.8 to 2.6)	0.72
Abdominal overweight	Education	52058	7.0 (6.2 to 7.8)	<0.0001	−2.3 (−4.5 to −0.1)	0.04
	Social class	50976	8.2 (7.4 to 9.0)	<0.0001	−3.2 (−5.4 to −1.0)	0.01

1 Estimated from binomial regression model for each prevalence measure and including SEP indicator, age as polynomial terms and four survey periods (1993/96, 1997/00, 2001/04, 2005/08): a positive value indicates that the prevalence is higher in the lower SEP subgroup.

2 Estimated from binomial regression model for each prevalence measure and including SEP indicator, age as polynomial terms, survey periods and interaction between four survey periods and SEP indicator: a positive value indicates that the difference in prevalence between SEP subgroups has widened over time.

3 Generalised obesity and overweight based on categories of body mass index.

4 Abdominal obesity and overweight based on categories of waist circumference.

5 Waist circumferences were not collected on the HSE core samples in 1995-96, 1999-00 & 2004.

6 Shorter vs longer time in education.

7 Manual vs non-manual social class.

The differences in prevalence between SEP groups when comparing 1993/6 to 2005/8 varied between decreasing by 2.7% and increasing by 3.0% over time across the outcomes: where a positive value indicates that the difference in prevalence between SEP subgroups has widened over time. With one exception, there was little evidence of significant widening or narrowing of inequalities over time between the SEP subgroups. The disparity in prevalence between education groups significantly increased for generalised obesity (P<0.0001): the difference was 3.0% higher in 2005/8 than it was in 1993/6 (95% CI 1.4 to 4.6%).

Among women, the prevalence of all outcomes were raised in the lower compared to the higher SEP groups averaged across the study periods (difference in prevalence varying between 6.0% and 9.7%) and these were all statistically significant. There was a slight tendency for the difference to be greater when comparing SEP groups defined by social class rather than education, though no formal comparisons were made. The differences in prevalence between SEP groups when comparing 1993/6 to 2005/8 ranged from decreasing by 3.2% and increasing by 2.7% across the outcomes. With one exception, there was little evidence of significant widening or narrowing inequalities over time between the SEP subgroups. The disparity in prevalence between education groups significantly increased for generalised obesity (P<0.0001): the difference was 2.7% higher in 2005/8 than it was in 1993/6 (95% CI 1.2 to 4.3%).

## Discussion

### Summary of Main Findings

The crude and age-adjusted prevalence of generalised and abdominal overweight and obesity increased markedly between 1993/4 and 2007/8 in men and women aged 18–75 years in England. There were statistically significant differences in the four outcomes in men and women across the two measures of SEP, except for social class differences in both generalised and abdominal overweight in men. In all cases, the prevalence of obesity and overweight was higher in subgroups with lower SEP (differences of 0.2% to 9.5%). There was limited evidence that the socioeconomic patterning of indicators of greater body fat has changed since the early 1990s. However, there were no consistent findings across the different outcomes and SEP indicators, despite a significant widening of the educational gradient over time for generalised obesity in men and women. Socio-economic gradients in outcomes were wider for relative education among men, and slightly wider for social class among women.

### Relationship to Existing Knowledge

There is a large literature on the associations between measures of obesity/overweight and measures of SEP, largely from single cross-sectional studies. A recent international review of studies on SEP and obesity found that the majority of studies in more developed countries reported associations between lower SEP and generalised obesity in women, though the associations were more likely to be non-significant in men. [4] This pattern was seen in this study and others in the UK [32]. Surveys in Britain have also found a social gradient in the prevalence of abdominal obesity among both men and women, though the evidence for this relationship in men was not always very strong. [33]–[36] These findings were echoed in a recent international review of educational inequalities in obesity and overweight in European countries. [37] This review also noted that countries in which the prevalence of overweight was very high tended to have differences in overweight across educational groups that were relatively small, and suggested that this may reflect that it is more difficult for the prevalence of overweight to increase much once a high level is attained. [37] However it is possible that all subgroups are likely to have a high prevalence if the overall prevalence is high, leaving less scope for inequality.

Inverse educational gradients were found to be a common phenomenon, particularly among women. It has also been noted that the strength of the relationship can vary with choice of SEP measure, and this feature has been seen in other UK-based studies [32], [38].

It has been suggested that the reason for the gender difference in strength of association may be that, although all receive the same health messages, there is a stronger emphasis on thinness in women, whereas men value a larger and more ‘muscular’ shape. [4] In addition, those with a higher SEP may be more likely to mix with others who value thin bodies and a healthy lifestyle. [4] Another reason for the gender difference came from a French study which found that both mean and ideal BMI were significantly inversely correlated with SEP in women only, while smoking prevalence was inversely correlated with SEP in men only. [39] It was suggested that a gender difference in weight concern could explain this pattern: on top of ideal body weight decreasing with SEP in women, women also report being more concerned about weight gain if they stop smoking, whereas men may be more concerned about the potential health gains from doing so and this view increases with SEP [39].

There have been fewer studies that have investigated whether the trends (as opposed to the cross- sectional pattern) in obesity and overweight differ between SEP subgroups. These are usually based on repeated cross-sectional designs and have not produced common findings. This is, perhaps, not surprising, since studies have varied in outcome measures (prevalence of obesity and/or overweight based on BMI or WC ), measures of SEP (educational level of participants or their parents, social class or income, individual occupation or that of head of household), self-reported or researcher-obtained measurements, time periods over which data were collected, countries in which surveys took place, study sizes, and differing approaches to statistical analyses.

Some have investigated a changing social gap in obesity or overweight without making any formal statistical assessment. A series of surveys of 18-year olds in Sweden over 30 years reported a widening education gradient, [14] as did a Swiss series of surveys of adults between 1992–2007, [19] and it was found that adult obesity had increased faster in manual than non-manual workers in France during 1981–2003. [7] A study in Spain concluded that the obesity gap by education status had widened in women but narrowed in men. [11] However, two studies of US obesity trends between 1984–1994 and 1971–2000 concluded that the educational advantage in obesity was declining, [12], [40] as did one concentrating on ethnic immigrant groups in the United States over 1976–2008, [21] and a study of young Belgian males between 1979–99 reported no change in socioeconomic inequality based on the median income of municipalities [18].

The studies in which formal statistical tests were carried out, or the strength of interaction estimated, also reported differing patterns. A significant widening of the social gradient was reported between income and obesity in Brazilian adults during 1975–2003. [20] This was also reported for occupation and obesity in France during 1995–2005 [6] and in Switzerland in men only during 1993–2000. [10] The education-obesity gradient was shown to have increased significantly in France during 1970–2003, [22] in Swiss men only during 1993–2000, [10] in Belgium for men only between 1997–2004, [18] and for women only in the United States between 1999–2008. [9] However, social inequality associated with education had decreased significantly in younger adults only between 1980–1997 in Sweden. [17] There were also a number of studies in Europe and the USA where, although there were some signs of changing trends, there were found to be no statistically significant changes in the SEP-obesity relationship. [8], [13], [15], [16], [18], [23], [24] A recent meta-regression of data in women aged 18–49 from 37 developing countries in 1998–2007 found that, in the majority of countries, higher SEP was associated with higher gains in the prevalence of overweight [41].

### Strengths and Limitations of the Methods

The HSE is the largest representative population health survey undertaken regularly in England and provides a rich source of data. However, there has been some inconsistency with which specific variables have been collected, and changes in the definitions of some variables from year to year limits the utility of these data to track trends. In particular, waist circumference was not collected in the HSE core sample for some years. There was a noticeable fluctuation in the proportions in social class groups in 2000, when the new Registrar General’s Standard Occupational Classification was introduced; it is unclear what effect this may have had on our findings. Although response rates to the HSE are relatively high, there is the possibility of non-response bias. Those in the lower socioeconomic groups may be less likely to respond. The methodological reports of the HSE compared the age and sex distribution of HSE participants with that from the national Census, [26] and have found that women and older people are slightly over-represented. The HSE introduced weighting for non-response in 2003, but these weights were only available for four of the years in this study and so have not been used. This also precluded a sensitivity analysis to explore the impact of non-response.

The average age of HSE participants aged 18–75 has increased over the study period, which will have contributed a little to the increased crude prevalence over time. However, it can be seen that the difference between crude and age-adjusted prevalence estimates are small. It was noted earlier that the proportion of those coded as ‘manual’ has declined over the period of the study: this reflects both a change in the occupational coding system in 2000 but also a decline in the jobs available which would be classified as ‘manual’. Nevertheless, the occupational coding used in this study splits the study population into a lower and higher SEP grouping which has been used in many other studies. There is no consistent method of classifying educational level in surveys, and few comparisons of any differences between various approaches. However, a recent analysis of data from OECD countries found that a model to predict obesity that includes the length of education relative to their peers, is preferred to one which includes the absolute length of education. [42] Given these findings, we have chosen to categorise length of education relative to their age-group. The school leaving age has increased over time, as has the opportunity for higher education, and this categorisation identifies participants who have a relatively long education for their age, and hence identifies those with a relatively high socioeconomic position in terms of their education, rather than simply the amount of education they had received. Nevertheless, even with this more complex coding, the proportion in the ‘lower’ subgroup has still decreased over time.

We have estimated the association between SEP and indicators of obesity from cross sectional data, but the direction of a causal link between SEP and adiposity requires longitudinal studies.

### Implications for Policy, Practice and Future Research

There is a need for policy and practice to focus on inequalities in obesity and develop interventions to reduce the gap between rich and poor. There is already a significant body of information, policies and action plans relating to obesity in the UK, much at national level, including the recent UK Foresight Obesity report. [43] Some of this makes specific reference to the social patterning of obesity, but more direct guidance on obesity interventions, such as from NICE, [44] does not acknowledge the potential importance of the social patterning for delivering interventions effectively and efficiently. Implementation of the NICE guidance on prevention and management of obesity, [44] as well as relevant national service frameworks (NSFs)[45]–[47] and implementation plans arising from existing and future government health strategies relating to obesity [46], [48]–[50] will need to take account of this social patterning and ensure that interventions proposed do not inadvertently further widen inequalities in obesity and overweight. [51] The recent Marmot review of health inequalities in England emphasised the importance of action to reduce the social gradient in health, and put forward a framework for the delivery and monitoring of these reductions. [52] This study has confirmed large socioeconomic inequalities in obesity and overweight, but found limited evidence that they are worsening. However, these findings should not be cause for complacency. Increasing prevalence of overweight and obesity, as well as wide inequalities in them, remain a significant cause for concern and will require extraordinary public health efforts over many years. [53] It is important these inequalities are studied further, as more data become available.
